# Biomass-Derived Carbon Dots from Guava Leaves Promote Rice Growth and Yield in a Dose-Dependent Manner

**DOI:** 10.3390/nano16120780

**Published:** 2026-06-20

**Authors:** Thi Xuan Phuong Tran, Petr Konvalina, Dang Hoa Tran, Xuan Diem Ngoc Le, Trong Nghia Hoang, Quoc-Bao Vo-Van, Duc An Hoang, Thanh Tien Do, Thanh Hai Duong, Dang Khoa Tran

**Affiliations:** 1 Faculty of Agronomy, University of Agriculture and Forestry, Hue University, 102 Phung Hung, Hue City 49000, Vietnam; ttxphuong@hueuni.edu.vn (T.X.P.T.); tdanghoa@hueuni.edu.vn (D.H.T.); 2 Faculty of Agriculture and Technology, University of South Bohemia in České Budějovice, Branišovská 1645/31a, 37005 České Budějovice, Czech Republic; konvalina@fzt.jcu.cz (P.K.); hoangn00@fzt.jcu.cz (T.N.H.); 3 Department of Electrical, Electronics and Materials Technology, University of Sciences, Hue University, 77 Nguyen Hue, Hue City 49000, Vietnam; lexuandiemngoc@hueuni.edu.vn; 4 Faculty of Engineering and Food Technology, Hue University, 102 Phung Hung, Hue City 49000, Vietnam; vvqbao@hueuni.edu.vn (Q.-B.V.-V.); dothanhtien@huaf.edu.vn (T.T.D.); 5 Faculty of Natural Sciences, Quy Nhon University, 170 An Duong Vuong, Quy Nhon City 61000, Vietnam; hoangducan@qnu.edu.vn; 6 Faculty of Animal Sciences and Veterinary Medicine, Hue University, 102 Phung Hung, Hue City 49000, Vietnam; duongthanhhai@huaf.edu.vn

**Keywords:** carbon dots, biomass-derived carbon dots, hydrothermal synthesis, rice, seed priming, grain yield

## Abstract

Biomass-derived carbon dots (CDs) have attracted increasing attention in agriculture due to their simple synthesis and low environmental impact. In this study, CDs were synthesized from guava (*Psidium guajava*) leaves using a hydrothermal method (200 °C, 15 h). The particles had an average size of 6.17 nm and a quantum yield of 2.46%, confirming the successful synthesis of fluorescent carbon nanomaterials from the natural precursor. The effects of CDs on rice (*Oryza sativa* L., variety HT1) were evaluated through both seed treatment and field application. Soaking seeds in a 200 ppm CD solution for 24 h significantly enhanced shoot and root lengths (28.87 mm and 34.00 mm, respectively) among the tested treatments. In field trials, applying CDs at the same concentration also promoted plant growth, as evidenced by improvements in plant height, leaf development, tillering, and flag leaf characteristics. These changes were reflected in yield, with the highest grain yield of 6.13 t ha^−1^ at 200 ppm, exceeding that of the control treatment. The observed positive effects may be due to enhanced photosynthetic activity and better control of oxidative processes in plants. Nevertheless, the effect was less pronounced at higher concentrations. This trend suggests a dose-dependent response.

## 1. Introduction

Rice is a staple food crop belonging to the Poaceae family of grasses, grown in many countries worldwide. It plays a vital role in global food security and in Asian countries. FAO statistics for 2023 revealed that the average rice yield was approximately 4.75 t ha^−1^, and the global cultivation area was about 168.36 million hectares [1]. In Vietnam, rice is a major food crop and the most important staple, with an average consumption of approximately 6.9 kg per person per month. What is more, Vietnam is one of the top three rice exporters globally. Nowadays, living standards are continuously improving; therefore, consumers are paying more attention not only to nutritional value but also to food safety.

To date, nanomaterials have been applied in numerous fields, including biology, industry, and agricultural research, due to their small size and outstanding properties. In agriculture, this trend is not only promoting a new cultivation method that respects the environment but also driving food safety production [2,3]. Carbon dots, an emerging member of the family of carbon nanomaterials, are defined as discrete spherical carbon particles with sizes typically smaller than 10 nm [4]. These CDs have a structure consisting of a core of sp^2^/sp^3^-hybridized carbon particles, either amorphous or crystalline, surrounded by functional groups such as carboxyl, hydroxyl, carboxylic acid, and amino, which form bonds with biological entities [5,6,7]. Thanks to these functional groups, CD materials have strong luminescence and are easy to manipulate. To synthesize carbon nanoparticles, various approaches are used, namely the wet chemical method, the hydrothermal method, the ultrasonic technique, and the microwave technique. Furthermore, the precursors used to synthesize CDs are extremely varied, ranging from synthetic chemicals to renewable biomass sources. The biomass sources for producing carbon dots can be various vegetables, various types of organic waste and agricultural by-products, and any part of the plant. Specifically, organic materials like citric acid, glucose, chitosan, fruit juice, rice, etc., organic by-products like banana peel, citrus peel, plant waste, rice husk, wheat straw, etc., or various plant parts like leaves, roots, stems, fruits, flowers, and seeds are seen as potential precursors to fabricate CDs [8,9,10,11,12].

In the last decade, nanotechnology has been recognized as promising approach to improve agricultural production. Currently, nanotechnology is applied in many fields, including biological imaging, drug and gene delivery, optical and thermal sensors, solar cells, and catalysts for degrading organic pollutants [13,14]. In agriculture, studies have indicated that nanomaterials can promote plant growth, improve plant performance under stress conditions, and increase agricultural productivity [15,16]. The potential benefits extend beyond productivity. Nanomaterials may also support more sustainable agricultural systems by improving the efficiency of agricultural inputs and reducing environmental impacts [17].

Among the different types of nanomaterials, carbon dots (CDs) are comparatively safer nanoparticles for agricultural applications [13,14]. Carbon nanomaterials are used not only as fertilizers and pesticides to boost crop yield and quality [18,19] but also to enhance plant resistance, improve soil, and reduce waste [20,21,22]. What is more, nanomaterials have attracted considerable attention as plant biostimulants because they can stimulate plant development and enhance crop performance [23]. Recent studies have shown that CDs can influence several important physiological processes, including seed germination, nutrient uptake, photosynthesis, and water transport during different stages of plant growth [3,24,25,26,27]. Recent evidence further suggests that CDs can boost plant development via multiple mechanisms. Treatment with CD increased the photosynthetic activity and the antioxidant responses in cotton plants [28]. Studies on melons also indicated that CDs influence several biological processes related to plant growth and development [29]. Lettuce garnered similar findings. Foliar application of CDs enhanced plant development and yield, as demonstrated by Tran et al. (2015) [30]. These findings highlight the potential of CDs as nanobiostimulants for sustainable crop production.

Although carbon dots have attracted growing attention in agriculture, several questions remain unanswered. Most studies have been conducted under controlled laboratory or greenhouse conditions, while field experiment data remains limited. Moreover, carbon dots derived from plant-based materials have been less studied than those from conventional sources, and their effects on crop performance remain poorly understood. Rice responses to carbon dots can also vary with their characteristics and application rates, but comparisons across different concentrations remain relatively scarce. As a result, there is still limited evidence on whether plant-derived carbon dots can be used effectively to enhance rice growth and yield under real farming conditions.

Based on the unique optical and surface properties of carbon dots (CDs), we hypothesized that CDs derived from guava leaves could function as nanobiostimulants to enhance rice growth and yield by improving physiological activity and resource utilization. We further speculated that the extent of these effects would be influenced by the concentration of CDs, which leads to an optimal dosage range. To test these hypotheses, we characterized the CDs synthesized from guava leaves and applied them to rice under both germination and cultivation conditions. These findings aim to offer new insights into the potential of biomass-derived nanomaterials as sustainable tools for boosting rice production.

## 2. Materials and Methods

### 2.1. Materials

Fresh leaves of guava (*Psidium guajava*), both mature and juvenile, were collected from VietGAP (Vietnamese Good Agricultural Practice) orchards in Kim Tra Ward, Hue City, Vietnam. These leaves were the third from the apex, light green, and fully developed morphologically. Leaves were chosen as a biomass precursor because of their availability, low cost, and high carbohydrate content (43.18%) [31], which provides functional groups for the synthesis of carbon dots.

### 2.2. Synthesis of Carbon Dots (CDs)

**Figure 1:**
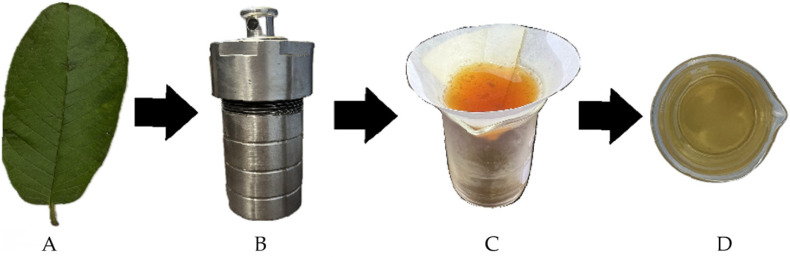
Process of fabricating CD materials from guava leaves. (**A**) Guava leaves; (**B**) Teflon-lined stainless steel autoclave; (**C**) Image of CDs in water solution under natural light; (**D**) Image of CDs in water solution under 405 nm ultraviolet irradiation.

The hydrothermal method used to fabricate the CDs solution is described as follows: Guava leaves are washed with distilled water and cut into approximately 5 × 5 mm pieces. Weigh 3.0 g of guava leaves and then disperse them into 80 mL of double-distilled water. The obtained mixture was sealed in a Teflon-lined autoclave (100 mL capacity) and heated from ambient temperature to 200 °C at 7 °C min^−1^, then maintained at that temperature for 15 h. After hydrothermal treatment, the entire autoclave is cooled to room temperature. The resulting solution was purified using filter paper and centrifuged at 14,000 rpm for 15 min to remove the larger sediment. The final solution obtained, CDs, brownish-yellow, homogeneous, and light with a pleasant smell, was stored at 4 °C, ready to use. The entire fabrication process of the CDs solution is schematically summarized in Figure 1.

### 2.3. Characterization of Carbon Dots

Morphology and particle size were determined using transmission electron microscopy (TEM, JEOL JEM-1010, JEOL Ltd., Tokyo, Japan) at an acceleration voltage of 100 kV. Particle diameter was measured in ImageJ (1.54p 17 February 2025), and average particle size was calculated in Origin.

For Fourier transform infrared (FTIR) spectroscopy analysis, a small amount of the carbon dot solution was deposited onto a KBr substrate and allowed to dry naturally before measurement using an FTIR Affinity-1S spectrometer (Shimadzu, Kyoto, Japan). For the optical measurements, the original carbon dot solution was diluted with double-distilled water to a series of concentrations, ensuring that the absorbance at 340 nm remained below 0.1.

UV–Vis absorption spectra were recorded using a GENESYS 10S spectrophotometer (Thermo Scientific, Waltham, MA, USA), and photoluminescence spectra were measured using an FS5 spectrofluorometer (Edinburgh Instruments, Livingston, UK). The quantum yield of the carbon nano solution is calculated using the comparative method based on the following expression [32]:

(1) $$QY={QY}_{R}\left(\frac{m}{m_{R}}\right)\left(\frac{n^{2}}{{n_{R}}^{2}}\right)$$

where *QY* is the quantum yield of the carbon solution; *QY*_R_ is the quantum yield of quinine sulfate as a reference, *QY*_R_ = 0.54; *m* and *m*_R_ are the slopes of the calibration curves obtained from the relationship between absorbance and integrated photoluminescence (PL) intensity of the sample and reference, respectively; and *n* and *n*_R_ are the refractive indices of their corresponding solvents (*n* = *n*_R_ = 1.33).

Quinine sulfate prepared in 0.1 M H_2_SO_4_ was used as the standard, and the synthesized carbon dots were dispersed in double-distilled water. Both solutions were diluted to a low concentration so that their absorbance at 340 nm was <0.1 [24]. Their photoluminescence spectra were then measured using 340 nm excitation, and the emission intensity was obtained from the area under the spectra between 360 and 700 nm. Draw the calibration curve describing the relationship between absorbance and the area under the photoluminescence spectrum curve of the carbon nano-solution and the quinine sulfate solution. After that, the quantum yield is determined by using Formula (1).

### 2.4. Seed Germination Experiment

The germination experiment was conducted at the Faculty of Agronomy, University of Agriculture and Forestry, Hue University, Vietnam. A split-plot design was applied with carbon dot concentration (100–600 ppm) as the main factor and soaking duration (12, 24, and 36 h) as the sub-factor.

Rice HT1 seeds (moisture content 13–15%) were pretreated in warm water (50–54 °C) for 10 min and subsequently soaked in CD solutions at the specified concentrations of 100 ppm, 200 ppm, 300 ppm, 400 ppm, 500 ppm, and 600 ppm for three time periods of 12 h, 24 h, and 36 h. The soaking solution was renewed every 12 h. Control treatment is N0 using reverse osmosis (RO) water. Each treatment included three replicates with 30 seeds per replicate. After treatment, seeds were incubated under controlled conditions. Monitoring parameters: germination percentage; seedling stem length (mm); and seedling root length (mm).

### 2.5. Field Experiment

The field experiment aims to assess the impact of varying concentrations of CDs solution on the growth and development of the HT1 rice variety.

Field experiments were conducted from May to September 2024 in Huong An Ward, Huong Tra Town, Thua Thien Hue Province, Vietnam. HT1 seeds were sown under eight treatments: a treatment N0 (no chemical fertilizer and no CDs), a control treatment N (with fertilizer and no CDs), and six water-soluble CD treatments (CDs at 100, 200, 300, 400, 500, and 600 ppm).

The recommended fertilization regime was applied in accordance with the prescribed dose by local agriculturists, consisting of 100 kg N ha^−1^, 65 kg P_2_O_5_ ha^−1^, 70 kg K_2_O ha^−1^, and 500 kg lime ha^−1^. During the experiment, the ATMOS-41 weather station recorded daily weather conditions, with the highest temperature being 32 °C, the lowest being 24 °C, the average temperature being 28 °C, and the average monthly rainfall being 150 mm. The temperature averaged 28 °C, ranging from a minimum of 24 °C to a maximum of 32 °C, while the mean monthly rainfall and humidity were 150 mm and 82%, respectively.

The experiment was arranged in a randomized complete block design (RCBD) with three replicates and a plot size of 30 m^2^ (5 × 6 m). Rice plants were grown at a spacing of 20 × 20 cm with one plant per hill.

Fertilizers were applied as basal (100% lime, 100% P_2_O_5_, 20% N) and topdressing (10 days after transplanting: 10% K_2_O + 20% N; 20 days after sowing: 40% K_2_O + 30% N; 20 days before flowering: 50% K_2_O + 30% N). Carbon dot solutions were applied in conjunction with fertilization and weeding treatments.

Growth and yield parameters were measured on 10 plants per replicate, including growth duration, plant height, number of leaves, total and effective tillers, flag leaf characteristics, and yield components.

The metrics for assessing the growth, development, and yield of rice plants encompass growth duration (days), leaf count (leaves), height (cm), total tiller count (tillers), effective tiller count (tillers), flag leaf length, flag leaf width (cm), flag leaf area (cm^2^), yield, and yield components.

### 2.6. Statistical Analysis

Data was analyzed by using Statistica program (version 13.2, StatSoft Inc., Tulsa, OK, USA). Analysis of variance (ANOVA) was performed to assess the effects of different factors and their interactions on the measured parameters. Treatment means were compared using Tukey’s Honest Significant Difference (HSD) post hoc test at *p* < 0.05.

## 3. Results and Discussion

### 3.1. Synthesis, Structure, and Optical Properties of Carbon Dots

**Figure 2:**
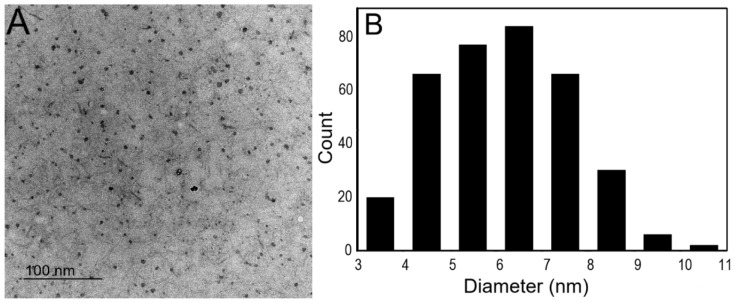
(**A**) TEM image with the scale bar of 400 nm. (**B**) The size histogram of CDs.

The TEM image of the prepared carbon dots (CDs) from guava leaves, generated by the hydrothermal method at 200 °C for 15 h, is shown in Figure 2A. The data indicate that the carbon nanoparticles are tiny and well diffused. The software ImagineJ (version 1.54d, National Institutes of Health, Bethesda, MD, USA) was used to quantify particle diameter, and a histogram was generated to statistically illustrate the particle size distribution in Figure 2B. This figure indicates a relatively broad distribution ranging from 3.46 to 12.05 nm, with an average diameter of 6.17 nm. This is similar to earlier investigations of biomass-derived CDs synthesized via hydrothermal methods [33,34,35].

**Figure 3:**
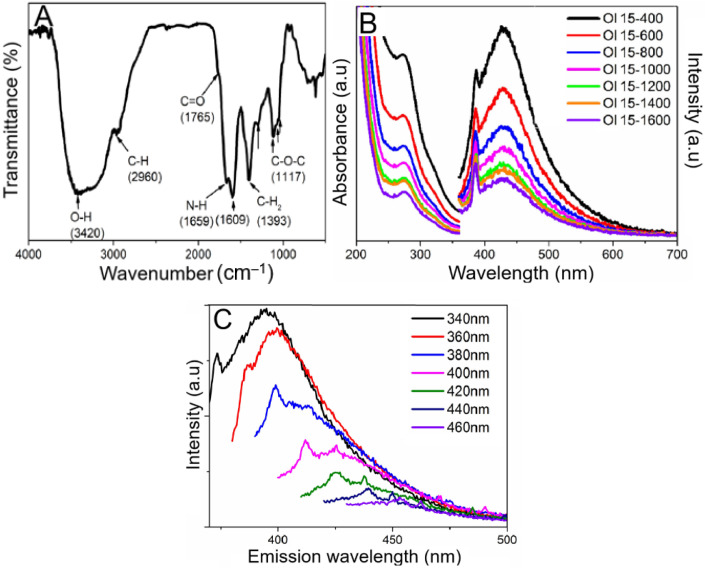
(**A**) Fourier transform infrared spectrometer spectrum. (**B**) Absorption spectrum (left side of the figure) and photoluminescence spectrum (right side of the figure) of the CDs solution, and (**C**) the PL spectra of the obtained CDs with varied excitation wavelengths.

The FTIR spectrum of the synthesized CDs (Figure 3A) displays a broad absorption band at around 3420 cm^−1^, which is characteristic of O–H stretching vibrations. The absorption peak at 2960 cm^−1^ is attributed to C–H bonds, whereas the peaks at 1765 cm^−1^ and 1659 cm^−1^ are associated with the C=O and N–H functional groups [33,36,37]. The peak at 1393 cm^−1^ is associated with C–H_2_ bending vibrations [37]. The peaks at 1300, 1240, 1071, and 1049 cm^−1^ are assigned to asymmetric stretching vibrations of C–O–C groups [38]. These results indicate that functional groups, including hydroxyl, carbonyl/carboxyl, and amino groups, were successfully introduced onto the surface of CDs during the hydrothermal process.

The UV–Vis absorption spectrum in Figure 3B depicted a high peak at approximately 280 nm, which corresponds to the n–π* transition of C=O bonds [39]. Photoluminescence (PL) analysis showed excitation-dependent emission behavior, with the emission peaks shifting from 395 to 455 nm as the excitation wavelength increased from 340 to 460 nm (Figure 3C). This is attributed to the heterogeneity of surface states and emissive traps, which depend on size, and resulting from the hybridization of the carbon core with surface functional groups. Such excitation-dependent fluorescence is a characteristic feature of CDs and is intimately connected to their potential role in light harvesting and energy transfer in biological systems.

**Figure 4:**
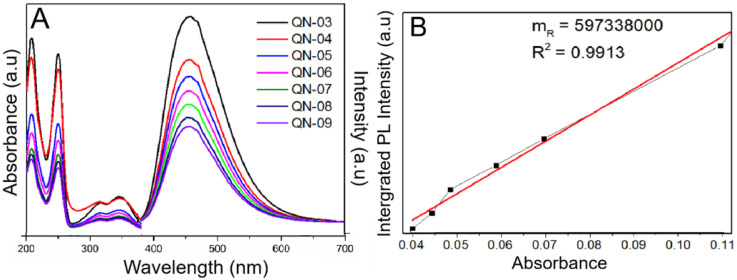
(**A**) Absorption spectrum (left side of the figure) and emission spectrum (right side of the figure). (**B**) The standard curve illustrates the correlation between absorbance and the area under the photoluminescence spectrum.

The PL spectrum measurement results are depicted in Figure 4A, and Figure 4B illustrates the slope of the relationship between absorbance and the area under the photoluminescence spectrum curve of the Quinine sulfate (QN) solution. Similarly, the correlation between absorbance and the area under the photoluminescence spectrum was examined for CDs solutions derived from guava leaves diluted to varying concentrations. The quantum yield (QY) of the CDs was determined to be 2.46% using quinine sulfate as a reference. The value is moderate but within the typical range of biomass-derived CDs and suggests the presence of active emissive centers.

The positive effects observed in this study may be partly related to the natural composition of guava leaves. Carbohydrates, amino acids, polyphenols, and other carbon-rich compounds present in the leaves contribute to the formation of carbon dots and may influence their surface characteristics. FTIR analysis confirmed the presence of hydroxyl, carbonyl, and amino groups, which may affect interactions between carbon dots and plant tissues. However, because only guava leaf-derived carbon dots were evaluated, it is not possible to determine whether these effects are unique to this biomass source. Future studies comparing carbon dots produced from different plant materials under the same conditions would help clarify how the choice of precursor influences carbon dot properties and plant performance.

### 3.2. Effects of Carbon Dots on Rice Variety HT1

#### 3.2.1. Effects of Carbon Dots on Seed Germination

Seed germination is an important developmental stage in seedling formation and crop production. The results of this study clearly showed that seed priming with carbon dots (CDs) synthesized from guava leaves improved germination and early seedling growth in HT1 rice. Results demonstrated an increase in germination percentage of 1.11–5.56% compared with all samples treated with the control treatment (RO), indicating a consistent stimulatory effect. However, differences in germination percentage among treatments were not statistically significant (*p* > 0.05), indicating that CD application had only a limited effect on germination.

**Figure 5:**
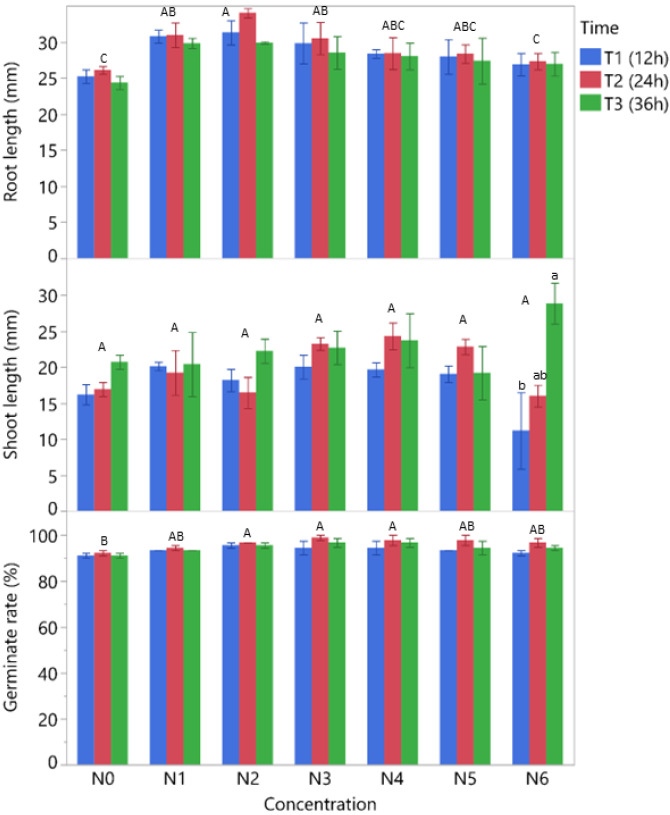
Effects of guava leaf-derived carbon dots (CDs) at different concentrations (N0–N6) and treatment durations (T1: 12 h, T2: 24 h, and T3: 36 h) on the root length (mm), shoot length (mm), and germination rate (%) of rice. Data are presented as mean ± standard error (SE) (n = 3). Different uppercase letters (A, B, C) indicate statistically significant differences among concentrations (*p* < 0.05). Different lowercase letters (a, b) represent significant differences among treatment durations within the same concentration (*p* < 0.05).

In contrast to germination percentage, which showed only limited variation across treatments, seedling growth parameters, especially shoot and root length, were more strongly affected by CD application. Statistical analysis indicates that shoot and root length were significantly affected by different concentrations of CDs (200–600 ppm) and soaking durations (12–36 h; *p* < 0.05). The result indicates that early seedling growth is more sensitive to the CDs’ treatment than the germination process (Figure 5).

**Figure 6:**
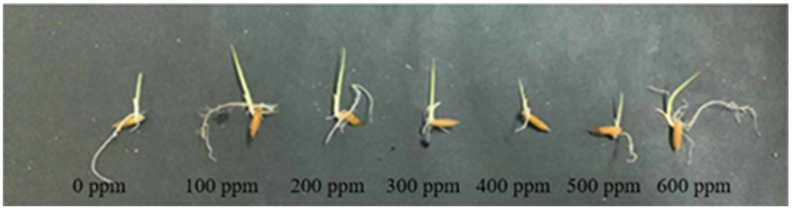
The effect of the CD solution concentration and seed treatment time (24 h) on the germination ability of the HT1 rice variety.

The maximum response was obtained at a 200 ppm concentration with a 24 h soaking duration, when shoot and root lengths were the longest (28.87 mm and 34.00 mm, respectively). This result confirms CD as a biostimulant in a dose- and time-dependent manner, with a clear optimal threshold above which no further improvement is noticed (Figure 6).

Their nanoscale physicochemical properties can explain the promotion of germination and seedling development by CDs. CDs can enter the seed coat through intercellular spaces and accumulate in the cotyledon, thereby promoting metabolic activation of the seed during germination. After radicle formation, CDs may be adsorbed onto the root surface and then enter the vascular tissues, allowing them to be transported from roots to shoots via the xylem. This systemic movement contributes to the development of shoot and root systems [3]. A mechanism of this kind would account for the relatively modest improvement in germination and the more marked enhancement of seedling growth.

The present findings partially agree with previous studies reporting beneficial effects of CDs on seed germination and early plant development [40,41]. Although the germination percentage was numerically higher in CD-treated seeds, the differences among treatments were not statistically significant. In contrast, significant improvements were observed in shoot and root growth, indicating that the primary effect of CDs was on post-germination seedling development rather than the germination process itself. Similar responses have been reported for Spirulina-derived CDs, which enhanced root growth and seedling performance following seed treatment [42]. The magnitude of improvement in the present study was greater, but these differences may be attributed to differences in the precursor materials for CDs, their particle characteristics, and experimental conditions.

Also, CDs synthesized from rice husk at concentrations of 100–400 mg L^−1^ were found to promote shoot and root growth of various plant species, including mung bean, water spinach, and mustard greens [43], indicating that the growth-promoting properties of CDs are broadly applicable to plant systems.

Although CDs stimulated growth at optimal concentrations, at high concentrations (≥400–600 ppm), growth did not increase continuously. Otherwise, growth performance decreases, particularly under prolonged soaking conditions. This is a classic hormetic response curve, where low-to-moderate doses stimulate biological activity, and higher doses may cause mild stress. Long-term exposure, especially at 36 h, can lead to excessive nanoparticle accumulation, perturbation of cellular homeostasis, and decreased growth efficiency. These results emphasize the necessity to optimize both concentration and exposure time for the use of CD in seed treatment.

Furthermore, the results also indicate a significant interaction effect of the CDs concentration and the soaking time. The best treatment combination (200 ppm, 24 h) was optimal only with this soaking duration, suggesting that the CDs’ effectiveness is governed by dose and exposure time. The shorter soaking duration (12 h) may not allow sufficient uptake of CDs to fully activate physiological processes. In contrast, the longer soaking period (36 h) may reduce the effectiveness of the CDs due to stress associated with overexposure. This interaction effect suggests that the uptake of CDs should be coordinated with plants’ physiological responses to achieve maximum benefit. Overall, the results confirmed that CDs are an efficient nano-biostimulant in seed priming, improving germination and early seedling growth of rice by improving physiological activation and internal transport processes. However, their effect is highly dependent on the interaction of concentration and soaking time. An optimum combination of these two factors is required to obtain the maximum biological response.

The findings of this study are consistent with previous research showing that nanomaterials can support plant growth and improve crop performance. Earlier studies have reported that these materials can influence how plants absorb and use resources, helping them grow more effectively under different conditions [15,16]. Their use in agriculture has therefore attracted considerable attention in recent years. Juárez-Maldonado et al. (2019) [23] noted that nanoparticles can stimulate plant growth and increase biomass production in several crop species. Research on carbon dots has also produced encouraging results. For example, Wu et al. (2026) [28] observed better growth in cotton after CD treatment, while Sousa et al. (2026) [29] found changes in biological processes linked to plant growth in melon. Although the present study did not directly examine these responses directly, the improvements observed in rice seedlings suggest that CDs may help plants use available resources more effectively during early development.

#### 3.2.2. Effects of Carbon Dots on Growth and Development of Rice

**Figure 7:**
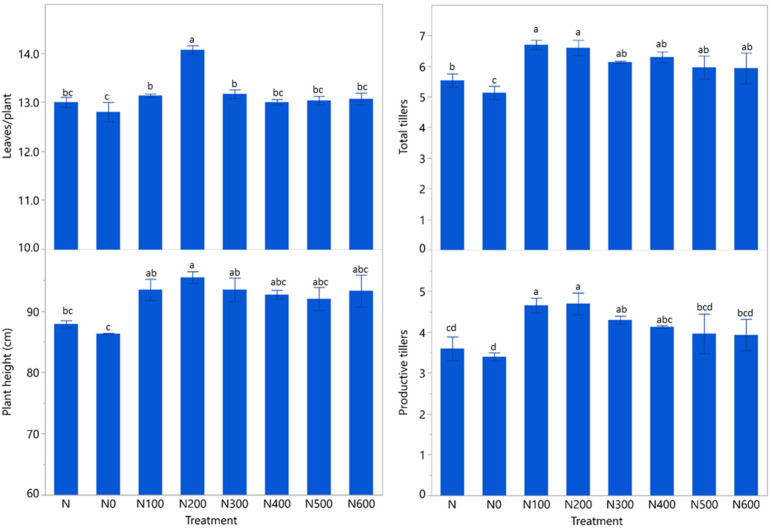
Effects of carbon dot (CD) concentration on growth parameters (plant height, number of leaves, number of tillers, and number of effective tillers) of HT1 rice. Values are means; different letters (a, b, c, and d) within each column indicate significant differences among treatments at *p* < 0.05 according to the HSD test.

Application of CDs significantly increased vegetative development in rice plants (Figure 7). Plant height, number of leaves, total tillers, and effective tillers were increased significantly (*p* < 0.05) compared to the control. The largest significant increase in plant height was 7.6–9.2 cm at 200 ppm.

Leaves are important organs for photosynthesis and biomass production. In this study, the number of leaves differed among treatments. The N-CDs200 treatment (14.07 leaves plant^−1^) achieved the highest leaf number, significantly differing from the other treatments (*p* < 0.05). The results indicate that CDs have a positive impact on vegetative growth via enhanced physiological activity.

The number of effective tillers, an important yield driver, was considerably higher in the treatments with 100–300 ppm CDs, with an increase of 0.9–1.3 tillers per plant over the control. The differences in the number of effective tillers among treatments were statistically significant (*p* < 0.05). The improvement is indeed significant as effective tillers directly contribute to panicle development and grain yield. CDs produce better photosynthetic performance, leading to increased growth.

Previous studies demonstrated that the application of CDs can improve the electron transport rate and photosystem II efficiency by 29.81% and 29.88%, respectively [44]. The physiological advantages of CDs are advantageous for carbon assimilation and biomass accumulation. In addition, CDs can be used as nano-enabled metabolic modulators of plants by enhancing nutrient absorption efficiency. CDs alleviate oxidative stress and help maintain cell integrity, thus providing an environment suitable for rice growth and development. Such improvements may enhance carbon assimilation and biomass accumulation, resulting in greater plant height, leaf production, and tillering.

**Figure 8:**
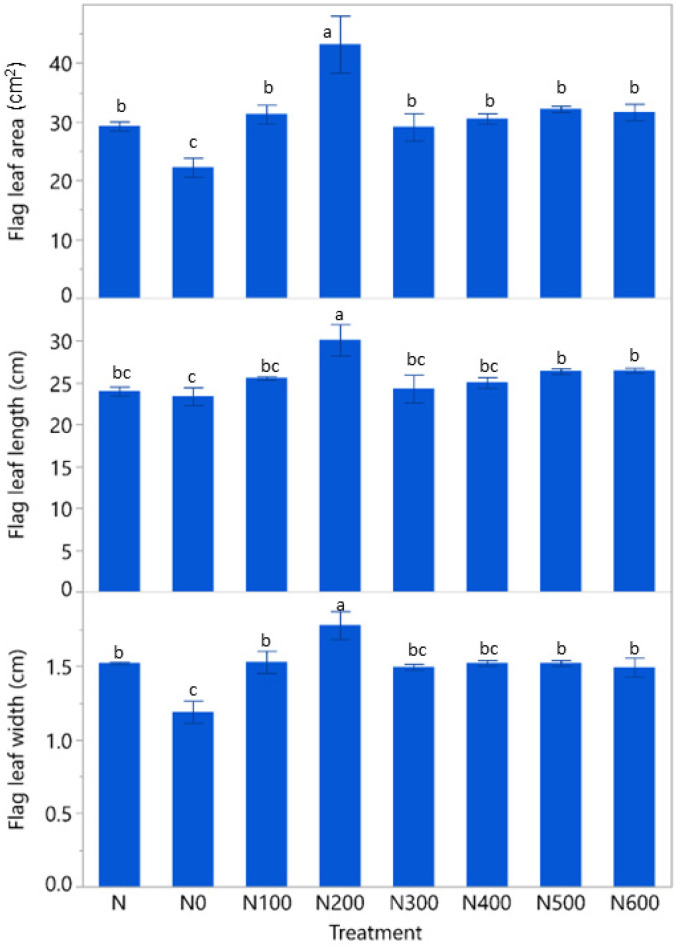
Effects of carbon dot (CD) concentration on flag leaf morphological traits of HT1 rice, including flag leaf length, width, and area. Data are presented as mean values. Different lowercase letters (a, b, and c) within the same column indicate statistically significant differences among treatments at *p* ≤ 0.05 based on the HSD test.

Flag leaf traits are important for yield formation, as this leaf can contribute about 50% of the assimilation required for grain filling. The results (Figure 8) show that the flag leaf length ranged from 23.37 to 30.12 cm and the width ranged from 1.19 to 1.78 cm. The flag leaf area ranged from 22.24 to 32.18 cm^2^, and the CDs treatment had a significantly higher leaf area than the control (*p* < 0.05)**.** These results indicate that the application of CDs stimulates plant growth by increasing leaf development and photosynthetic potential. The improvements in flag leaf development, therefore, may contribute directly to the enhanced productivity observed in CD-treated plants.

The positive effects of CDs on vegetative growth observed in the present study agree with recent reports on other crops. Wu et al. (2026) [28] demonstrated that CDs enhanced cotton growth through increased photosynthetic performance and antioxidant enzyme activity. Similarly, metabolomic analyses revealed that CDs altered metabolic pathways associated with plant development and carbon metabolism in melon [29]. Although these physiological responses were not directly measured in the present study, the significant increases in plant height, leaf development, tiller formation, and flag leaf area suggest that CDs may promote rice growth through similar physiological and biochemical processes.

#### 3.2.3. Effects of Carbon Dots on the Yield of Rice

**Figure 9:**
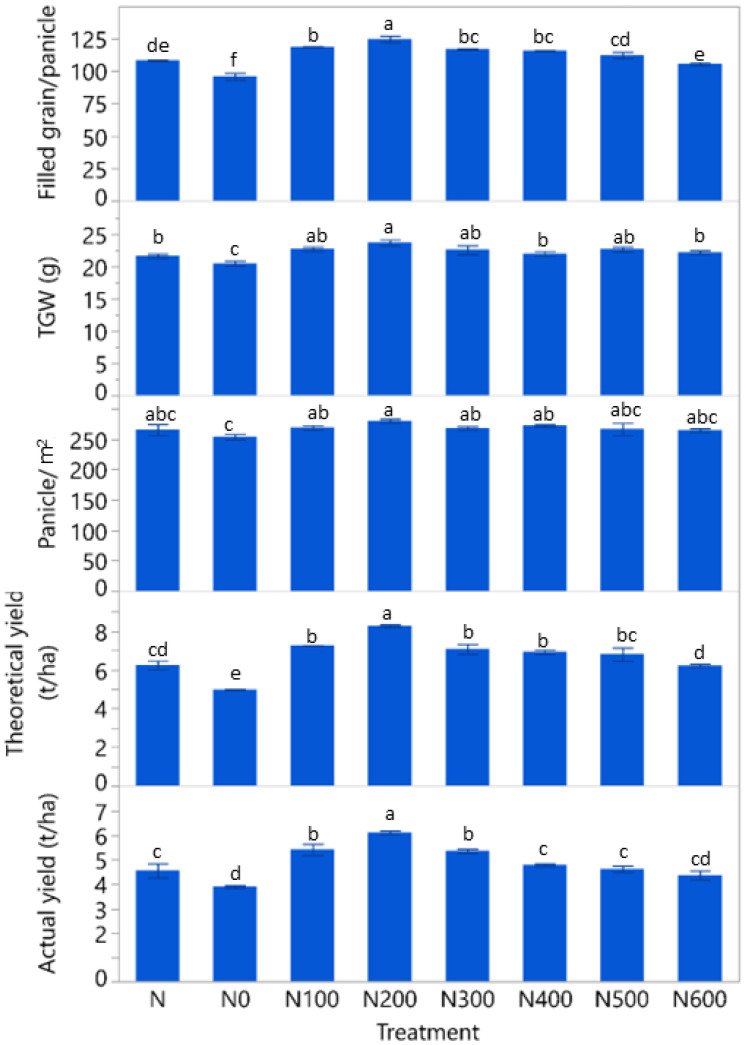
Effects of carbon dot (CD) concentration yield and yield components in experimental formulas, including panicles per m^2^, the number of filled grains per panicle, the thousand grain weight, theoretical yield, and actual yield. Data are presented as mean values. Different lowercase letters (a, b, c, d, e and f) within the same column indicate statistically significant differences among treatments at *p* ≤ 0.05 based on the HSD test.

Figure 9 represents the number of panicles per m^2^, the number of filled grains per panicle, the theoretical yield, and the actual yield of rice under different CD treatments. The number of panicles per m^2^ differed significantly among treatments (*p* < 0.05) and ranged from 253.67 to 280.00. The highest value was observed at 200 ppm. The maximum value was found at 200 ppm (280.00), and the minimum was in the control (253.67), representing an increase of approximately 10.4% compared with the control. The difference between treatments indicates that CDs had a clear effect on tiller formation. However, at higher concentrations (≥300 ppm), the number of panicles slightly decreased compared with the optimal level, although it remained higher than or comparable to the control.

The number of filled grains per panicle was significantly different among treatments (*p* < 0.05) and ranged from 95.83 to 124.73. The maximum value was obtained at 200 ppm (124.73), followed by 100 ppm and 300 ppm, while the minimum value was observed in the control (95.83). These results suggest that the CD application significantly increased grain filling. However, a decreasing trend was observed at higher concentrations, with treatments at 400–600 ppm recording lower values than the optimal treatment, suggesting a possible limitation in CD efficacy at excessive levels.

The theoretical yield ranged from 4.98 to 8.28 t ha^−1^ and was significantly influenced by the CD treatments. The highest theoretical yield was obtained at 200 ppm (8.28 t ha^−1^), followed by 100 ppm and 300 ppm, whereas the lowest value was recorded in the control treatment (4.98 t ha^−1^). The differences among treatments were statistically significant (*p* < 0.05) owing to the combined effect of improved yield components. As with other parameters, the theoretical yield decreased at concentrations above 200 ppm. Yield is the most important indicator of crop production and reflects the plant’s overall performance, growth, and development.

Yield consists of three main components: number of panicles per unit area, number of filled grains per panicle, and 1000-grain weight. The results in Figure 9 show that all treatments with CD application had higher values of these components than the control. The highest yield was obtained at 200 ppm with an actual yield of 6.13 t ha^−1^, which was significantly higher than the control (3.90 t ha^−1^). This increase in grain production was associated with higher panicle number and greater grain filling. These answers imply that CDs had a favorable impact on source–sink connections throughout plant growth. The enhanced yield at 200 ppm could be due to higher photosynthetic efficiency, greater assimilate production, and better translocation of assimilates to growing grains. Previous studies have shown that CDs can enhance photosynthetic performance, RuBisCO (ribulose bisphosphate carboxylase oxygenase) activity, and carbon assimilation [41,44]. At the same time, foliar application of nanocarbon dots has been reported to increase rice grain yield by enhancing leaf photosynthesis and assimilate translocation [45].

The yield improvements observed in the present study are consistent with earlier reports showing increases of 14.8% [40] and 12.3–13.2% [45] following CD application. By absorbing CDs through their leaves, rice plants may utilize more efficiently and enhance yield. However, the decline observed at concentrations above 200 ppm further supports the dose-dependent nature of CD responses. Similar concentration-dependent effects have been reported in soybean and other crops, where moderate concentrations stimulated growth and yield more effectively than higher concentrations [27].

These results further reinforce the increased interest in agricultural nanotechnologies. Nanomaterials have been found to increase agricultural productivity and quality and to foster more sustainable production methods [17,46]. While the mechanisms of CD-induced growth are not yet fully understood, there is increasing evidence that CD can concurrently affect numerous processes in crops such as photosynthesis, antioxidant regulation, nutrient absorption and primary metabolism [28,29]. Further studies combining physiological, biochemical, metabolic and molecular techniques are needed to better understand the interaction between biomass-derived CD and rice plants and their contribution to improved growth and productivity.

## 4. Conclusions

In this study, carbon dots (CDs) were successfully synthesized from guava leaves using the hydrothermal method (200 °C for 15 h). The resulting particles had a small average size (6.51 nm) and a quantum yield of 2.46%, this confirms the successful production of fluorescent carbon nanomaterials from a renewable biomass source. The use of guava leaves as a precursor offers a simple, environmentally friendly approach to producing biomass-derived CDs.

The results showed that the CD application had a positive effect on rice growth from the early stage to field performance. Although germination percentage was not significantly affected by CD treatment, seedling growth was enhanced, with the greatest shoot and root development observed at 200 ppm after 24 h of seed soaking. In field conditions, applying CDs at the same concentration improved several growth traits, including plant height, leaf development, and tillering.

These improvements are reflected in the yield, with the highest grain yield recorded at 200 ppm (6.13 t ha^−1^), but the effect decreased at higher concentrations, suggesting that rice’s response to CDs is dose-dependent.

In summary, the findings indicate that CDs derived from guava leaves can be a promising material for improving rice growth and productivity. Their ability to improve plant growth and yield under cultivation conditions highlights the practical value of biomass-derived nanomaterials in agriculture. Nevertheless, further work is still needed to elucidate the physiological and biochemical mechanisms underlying the observed responses and to evaluate the performance of these materials across different environments, rice varieties, and management conditions.

## Data Availability

The original contributions presented in this study are included in the article. Further inquiries can be directed to the corresponding author.

## Abbreviations

The following abbreviations are used in this manuscript:

CDs	Carbon dots
TEM	Transmission Electron Microscopy
FTIR	Fourier transform infrared
UV–Vis	Ultraviolet Visible
PL	Photoluminescence
RO	Reverse Osmosis
RCBD	Randomized Complete Block Design
ANOVA	Analysis of variance
HSD	Honest Significant Difference
RuBisCO	Ribulose bisphosphate carboxylase oxygenase
